# A warming welcome? Belgium’s increasing suitability for *Aedes albopictus*

**DOI:** 10.1186/s13071-025-07119-w

**Published:** 2025-11-26

**Authors:** Daniele Da Re, Isra Deblauwe, Emmanuelle Inès Kern, Marie Hermy, Javiera Rebolledo Romero, Katrien Tersago, Veerle Versteirt, Birgit Dumez, Cyrelle Houtsaeger, Lieze Rouffaer, Olivier Beck, Wim Van Bortel

**Affiliations:** 1 Research and Innovation Centre, Fondazione Edmund Mach, San Michele All’Adige, Italy; 2 Center Agriculture Food Environment, University of Trento, San Michele All’Adige, Italy; 3 Institute of Tropical Medicine, Unit Entomology, Antwerp, Belgium; 4 Imperial College London, London, UK; 5 Service Epidemiology of Infectious Diseases, Sciensano, Brussels, Belgium; 6 Department of Care Flanders, Division of Preventive Health Policy, Brussels, Belgium; 7 Policy Department-Wildlife Disease Management, Agentschap Natuur en Bos, Brussels, Belgium; 8 Cellule Permanente Environnement-Santé (CPES), Public Service of Wallonia, Jambes, Belgium; 9 Department of Pathobiology, Pharmacology and Zoological Medicine, Ghent University, Merelbeke, Belgium; 10 Department Nature Management, Bruxelles Environnement, Brussels, Belgium; 11 Institute of Tropical Medicine, Outbreak Research Team, Antwerp, Belgium

**Keywords:** *Aedes albopictus*, Europe, Mechanistic model dynamAedes

## Abstract

*Aedes albopictus*, commonly known as the Asian tiger mosquito, is an invasive species of significant public health concern due to its ability to transmit dengue, chikungunya, and Zika viruses. Since arriving in Europe in the late 1970s, this mosquito species has progressively expanded its range, with Belgium marking an important step in its northward spread. The increasing frequency of its introduction raises the urgent question of whether *Ae. albopictus* could become permanently present in the country. This study investigates the potential for the establishment of *Ae. albopictus* populations in Belgium using a mechanistic model and assessing the likelihood of successful establishment based on simulated introduction events. Our results indicate that the probability of establishment is highest in Flanders, particularly under scenarios of early and multiple introductions during the summer months, even though the interannual climatic variability still plays a major role in establishment success. The establishment of *Ae. albopictus* in Belgium would introduce new epidemiological risks, as the species could facilitate the transmission of non-native viruses during particularly warm summers. While the transmission windows may remain limited compared to Mediterranean regions, continued introductions and climate change could expand these periods, increasing the likelihood of local outbreaks.

**Graphical Abstract:**

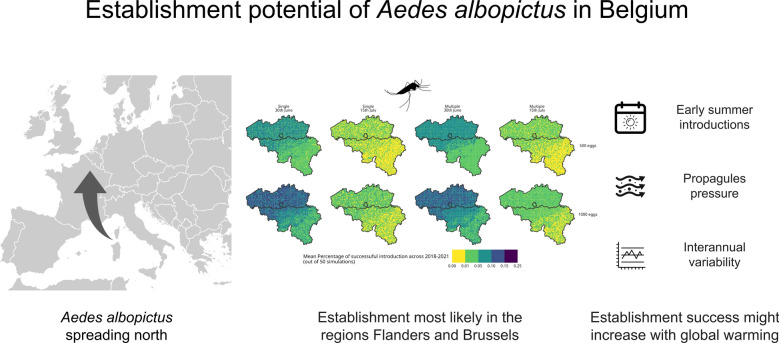

**Supplementary Information:**

The online version contains supplementary material available at 10.1186/s13071-025-07119-w.

Invasive mosquito species pose a major threat to global health by transmitting various pathogens to humans. One of the most notable invasive species in Europe is *Aedes (Stegomyia) albopictus* (Skuse, 1894) (Diptera: Culicidae), also known as the Asian tiger mosquito, which has been steadily spreading across the continent since its initial introduction in the late 1970s [1, 2]. This species has already been responsible for arbovirus outbreaks, mostly in Mediterranean Europe, making its establishment a significant public health concern. Native to Southeast Asia, *Ae. albopictus* has successfully spread to numerous European countries, including Italy, France, and Germany, due to global trade and transportation and favourable climatic conditions [3].

Invasive species follow a characteristic invasion curve, where early introductions are often hard to detect due to low population densities [4]. Over time, the population grows exponentially if left unchecked, making eradication efforts increasingly difficult and costly. Early intervention, when populations are still small and localised, provides the best opportunity for successful eradication. In Belgium, surveillance efforts such as the MEMO [Monitoring of Exotic Mosquitoes] and MEMO+ projects have been ongoing over the past decade, with a primary focus on detecting the introduction of *Ae. albopictus* and other invasive mosquito species [5]. These efforts have shown a marked increase in introduction events, particularly in the northern region of Belgium, Flanders. In 2023, overwintering activity was confirmed for the first time in the Flemish municipalities of Wilrijk and Lebbeke, marking a potential turning point in the species' invasion trajectory [6, 7]. In 2024, overwintering was observed in three additional locations [8] (Fig. 1). Given the continuous influx of propagules in the country and the first evidence of overwintering populations, the key question we now face is whether the increasing frequency of introductions will lead to the widespread establishment of permanent populations in this area situated at the edge of the current species’ invasive range.

**Fig. 1:**
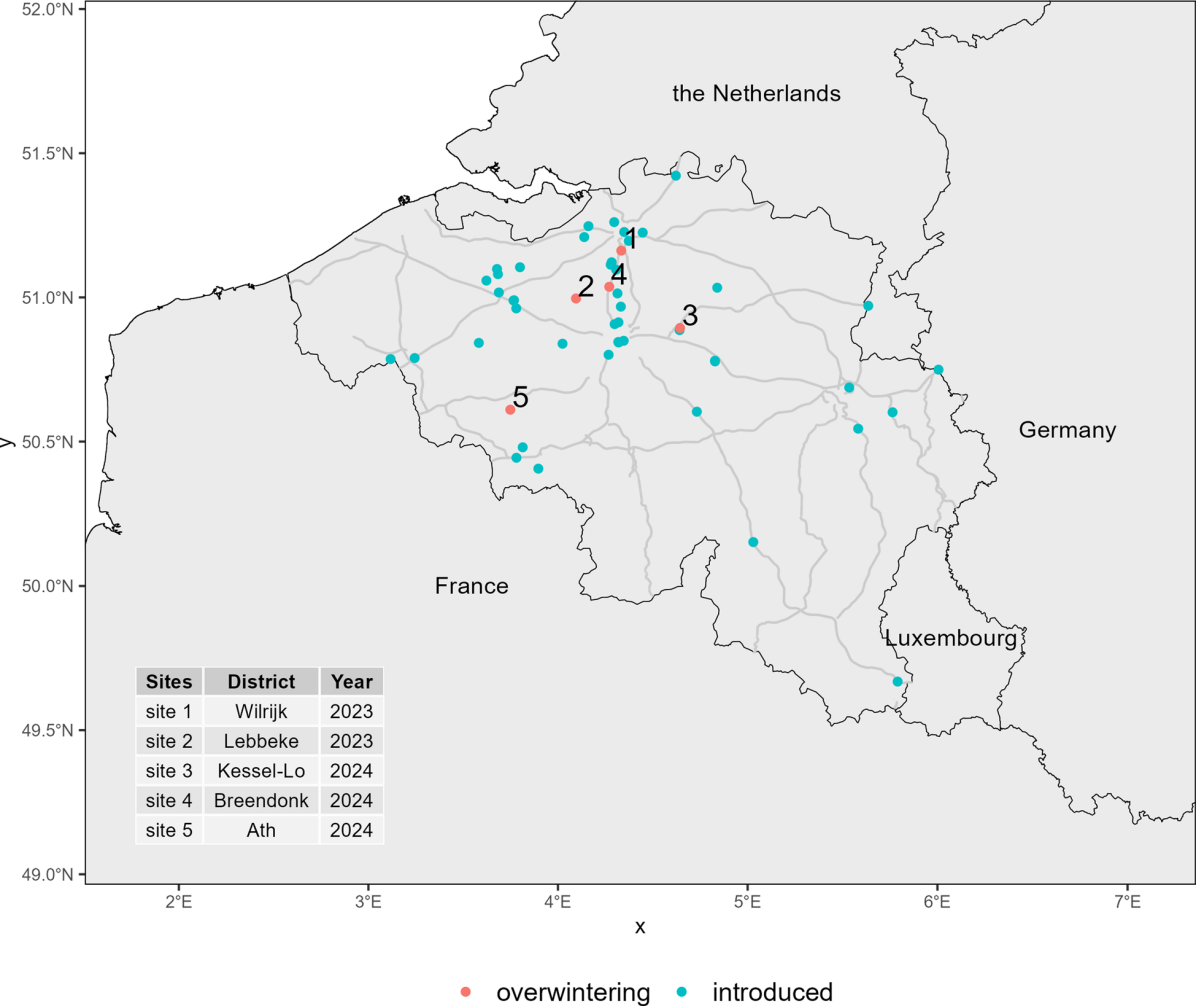
The introduction locations (blue) and overwintering observations (red) of *Aedes albopictus* in Belgium over the period 2000–2024. Inset: the year of observed overwintering at the five locations (districts). Country borders: black. Main routes: light grey

We used the dynamAedes [9] model to explore the likelihood of *Ae. albopictus* establishment in Belgium under different simulation scenarios. We simulated a single introduction event by introducing either 500 or 1000 eggs on June 30 or July 15 for the years 2018, 2019, 2020, and 2021, hence allowing us to consider the interannual variability in climatic conditions (Additional file 1: Table S1). We selected the egg introduction sizes based on the stochastic nature of the dynamAedes model, which limits the viability of establishment from very small founder populations (e.g., 1–5 adults), as these are highly likely to result in extinction due to demographic stochasticity. By introducing 500 and 1000 eggs as proxies for adult introductions, we aimed to reflect the average fecundity of *Ae. albopictus* females, which lay approximately 70 eggs per gonotrophic cycle [10]. These quantities correspond roughly to the reproductive output of 5–8 and 15–20 adult females, respectively, providing more statistically robust starting conditions for simulating potential establishment.

The introduction event occurred in all the pixels of a lattice grid of 2.5 × 2.5 km spatial resolution extending over the whole country. As this was a stochastic model, we repeated each introduction scenario 50 times. For each iteration and introduction scenario, the model was run for 1 year, after which we evaluated the probability of successful introduction, i.e., overwintering. This was measured as the proportion of dynamAedes model iterations that resulted in a viable population 1 year after the initial introduction event [9]. To reduce the influence of short-term fluctuations and better reflect sustained successful introductions, we averaged the percentage of successful introductions over the last 30 days of each simulation. This smoothing approach using a fixed temporal window reduced the impact of inherent stochastic variability and provided a more stable percentage of successful introduction metric.

In addition to these single introduction events, we simulated multiple introduction events. These consisted of an initial introduction of 500 or 1000 eggs on June 30 or July 15, followed by three random introductions of approximately 100 eggs each, continuing until September 15 of the same year (Additional file 1: Table S1). As with the single introduction events, we assessed the probability of successful introduction over the final 30 days of each simulation.

The dynamAedes model was informed using mechanistically downscaled temperature surfaces simulating mean daily air temperatures for 2018–2022 at high spatial resolution. These climatic surfaces were generated using the microclima R package [11] and derived by downscaling a regional climate model (from ERA5-Land [12] at 9 km spatial resolution) with a digital elevation model (DEM) at a 2.5 km spatial resolution. To enhance accuracy in urban environments, we also applied corrections for energy flux exchanges in built-up areas, accounting for the unique thermal dynamics of urban landscapes [11].

We quantitatively assessed the factors influencing the percentage of successful introductions across the different introduction scenarios using a generalised linear model (GLM) with a beta-binomial error distribution to account for overdispersion in the proportional count data. The model was specified as follows:

1 $${\text{Percentage of successful introduction }} \sim {\text{ introEggs}}\, + \,{\text{introMonth}}\, + \,{\text{introduction}}\, + \,{\text{introYear}}\, \times \,{\text{Region}},$$

where introEggs represents the number of eggs of the first introduction event, introMonth denotes the month of the first introduction event, introduction refers to the introduction type (single vs multiple), and the interaction term introYear × Region captures spatio-temporal variability across years and regions. The baseline levels for the categorical predictors were set as 2018 for introYear, Brussels for Region, and multiple introductions for introduction. Model selection was based on Akaike information criterion (AIC) comparison and likelihood ratio tests, which indicated strong support for the beta-binomial model over a standard binomial model (ΔAIC = 23,485). Model diagnostics using the DHARMa [13] package showed no evidence of zero inflation or residual outliers, slight underdispersion (dispersion = 0.96), and minimal deviation from uniformity, supporting the adequacy of the final model (Eq. 1).

Additionally, we investigated the factors influencing the simulated egg abundance across the different introduction scenarios. Each simulation produced a stack of rasters representing daily counts of the various life stages per pixel, from the date of introduction through the assessment period in the following year. Since not all introductions led to viable populations, we first calculated the daily egg abundance at the 95th percentile of simulated values. This approach filtered out low-abundance cases, allowing us to focus on scenarios indicative of successful introductions. The resulting daily values were then aggregated weekly to generate a smoothed and temporally consistent measure of egg abundance for each pixel. For each pixel, the total number of eggs observed over the assessment period was used as the response variable. To account for broad-scale spatial and temporal variability, we specified a GLM with the same structure and predictors described in Eq. 1:

2 $${\text{Total weekly egg abundance }} \sim {\text{ introEggs}}\, + \,{\text{introMonth}}\, + \,{\text{introduction}}\, + \,{\text{introYear}}\, \times \,{\text{Region}} .$$

Given the count nature of the response variable, we initially fitted the model using a Poisson distribution, but diagnostic checks indicated overdispersion. Therefore, we opted for a negative binomial GLM to better account for variability in the data.

Simulations using the dynamAedes model revealed that the success of *Ae. albopictus* introduction in Belgium is strongly influenced by both introduction dynamics, namely the timing and number of eggs, and spatio-temporal variability in climatic conditions (Fig. 2; Table 1). Single introduction events led to successful introductions in some cases, but success was generally limited and highly dependent on early-season introductions (e.g., by June 30), which allowed more time for population growth and egg accumulation before winter. Higher introduction sizes (e.g., 1000 vs 500 eggs) also improved the successful introduction probability. In contrast, scenarios with multiple introductions showed a slightly higher likelihood of overwintering, particularly when initial introductions occurred earlier and total egg input was greater.

**Fig. 2:**
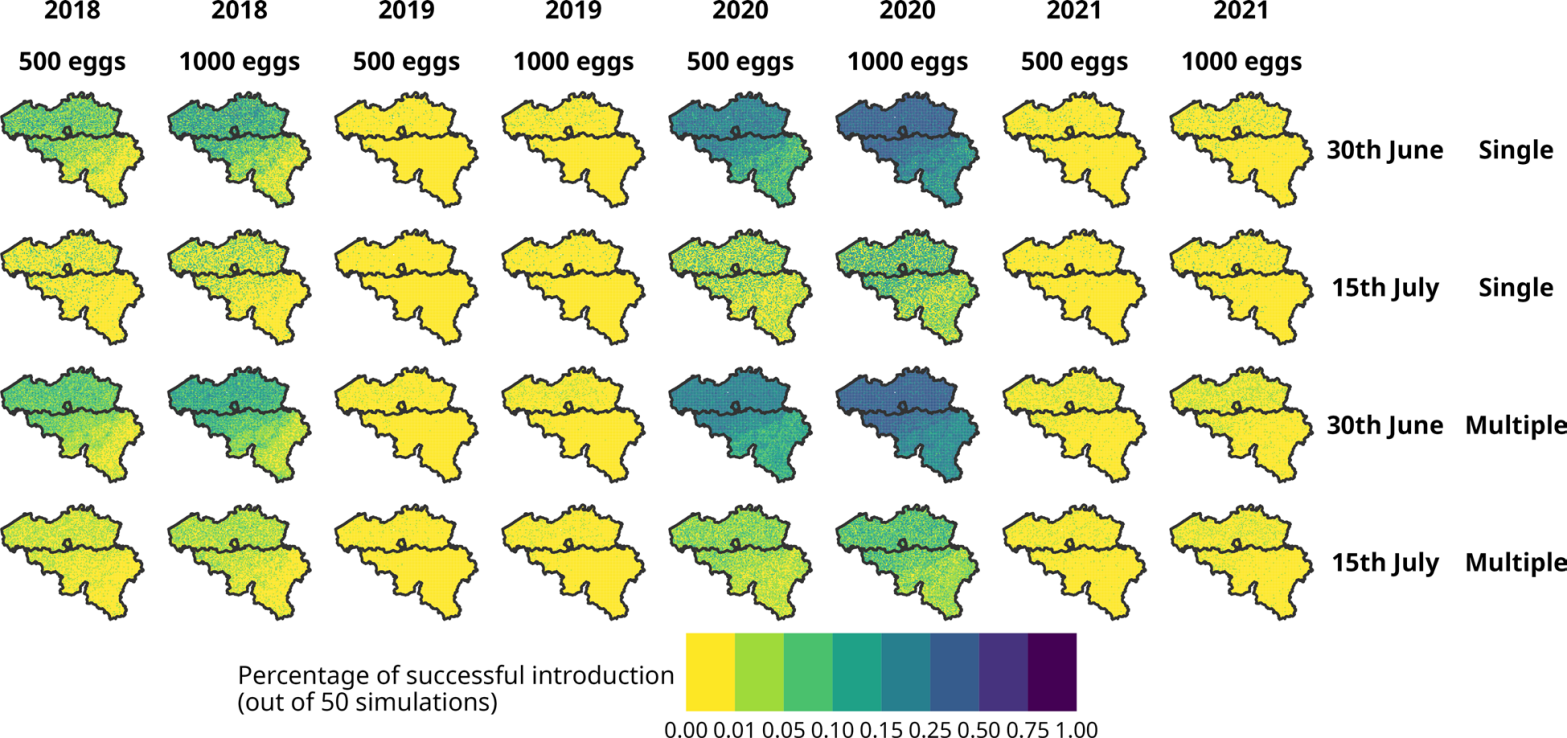
Percentage of successful *Aedes albopictus* introductions from 2018 to 2021 under scenarios involving single or multiple introduction events, starting with either 500 or 1000 eggs on June 30 or July 15 of each year. Values represent the average percentage of successful introductions over the final 30 days of each simulation. Lines represent the three regions of Belgium: Flanders (North), Wallonia (South) and Brussels (within Flanders)

**Table 1:** Parameter estimates from the beta-binomial generalised linear model (GLM) assessing factors influencing the proportion of successful introductions of *Aedes albopictus*. The model includes the number of eggs in the first introduction event (*introEggs*), month of the first introduction (*introMonth*), type of introduction (*introduction*, single vs multiple), and the interaction between year and region (*introYear* × *Region*) to capture spatio-temporal variability. Baseline levels for categorical variables are set to 2018 (*introYear*), Brussels (*Region*), and multiple introductions (*introduction*). Estimates are presented on the log-odds scale with associated standard errors (SE), *Z*-values, and *P*-values. The model was fitted using a beta-binomial distribution to account for overdispersion in the proportional response

Predictor	Estimate	SE	*Z*-value	*P*-value
(Intercept)	−2.63605	0.049094	−53.7	< 0.0001
introEggs 1000	0.574443	0.003956	145.2	< 0.0001
introMonth July	−1.677836	0.004758	−352.7	< 0.0001
Single introduction	−0.049376	0.003907	−12.6	< 0.0001
introYear2019	−4.467238	0.3814	−11.7	< 0.0001
introYear2020	1.251585	0.058313	21.5	< 0.0001
introYear2021	−3.053492	0.195284	−15.6	< 0.0001
RegionFlandres	0.037207	0.049231	0.8	0.4498
RegionWallonia	−0.918129	0.049431	−18.6	< 0.0001
introYear2019 × RegionFlandres	0.972428	0.382158	2.5	0.0109
introYear2020 × RegionFlandres	−0.013368	0.058602	−0.2	0.8195
introYear2021 × RegionFlandres	0.415163	0.195968	2.1	0.0341
introYear2019 × RegionWallonia	−0.223655	0.386191	−0.6	0.5625
introYear2020 × RegionWallonia	0.462309	0.058776	7.9	< 0.0001
introYear2021 × RegionWallonia	0.164771	0.196959	0.8	0.4028

These simulation findings were supported by the beta-binomial GLM, which identified significant positive effects of the number of eggs (*β* = 0.574, *P* < 0.001) and negative effects of later introduction months (*β* = −1.678, *P* < 0.001) and single introductions (*β* = −0.049, *P* < 0.001) on the probability of successful introduction. Spatial variation also played a role: Wallonia showed a significantly lower probability of successful introduction than the baseline region (Brussels), while Flanders did not differ significantly. Importantly, the interaction terms of the GLM model highlighted that the effects of time and space were not uniform, as the interactions were not always statistically significant (Table 1).

The GLM model on the simulated egg abundance dynamics also supported this interpretation, showing a positive and significant estimate for the introduction of 1000 eggs to the baseline of 500 eggs, and a negative and significant estimate for the later introduction date (15th of July vs 30th of June; Table 2). Single introductions also resulted in a negative and significant effect with respect to the baseline (Multiple).

**Table 2:** Parameter estimates from the negative binomial generalised linear model (GLM) assessing factors influencing total weekly *Aedes albopictus* egg abundance. The model includes the number of eggs in the first introduction event (introEggs), month of first introduction (introMonth), type of introduction (introduction, single vs multiple), and the interaction between year and region (introYear × Region) to capture spatio-temporal variability. Baseline levels for categorical variables are set to 2018 (*introYear*), Brussels (*Region*), and multiple introductions (*introduction*). Estimates are presented on the log scale with associated standard errors (SE), *Z*-values, and *P*-values. The model was fitted using a negative binomial distribution due to overdispersion detected in initial Poisson models

Predictor	Estimate	SE	*Z*-value	*P*-value
(Intercept)	11.504	0.0155	740.1	< 0.0001
introEggs 1000	0.497	0.001	475.94	< 0.0001
introMonth July	−0.34	0.001	−326.2	< 0.0001
Single introduction	−0.102	0.001	−97.78	< 0.0001
introYear2019	−1.344	0.0221	−60.93	< 0.0001
introYear2020	−0.456	0.0219	−20.78	< 0.0001
introYear2021	−3.287	0.0219	−149.8	< 0.0001
RegionFlandres	0.235	0.0156	15.08	< 0.0001
RegionWallonia	−0.937	0.0156	−60.15	< 0.0001
introYear2019 × RegionFlandres	−0.018	0.0222	−0.8	0.4235
introYear2020 × RegionFlandres	−0.018	0.0221	−0.83	0.4048
introYear2021 × RegionFlandres	0.03	0.0221	1.36	0.1740
introYear2019 × RegionWallonia	0.102	0.0221	4.6	< 0.0001
introYear2020 × RegionWallonia	0.04	0.022	1.79	0.0728
introYear2021 × RegionWallonia	0.032	0.022	1.46	0.1445

To complement the analysis of overwintering success, we investigated the spatial and temporal variability in air temperatures across the same pixels and time period. An analysis of variance (ANOVA) revealed significant effects of season, year, region, and their interaction on temperature (*P* < 0.001 for all factors; Additional file 1: Table S2), confirming pronounced spatio-temporal heterogeneity. Post hoc Tukey comparisons further demonstrated consistent temperature differences between regions, seasons, and years, with significant pairwise differences detected within each season and across regions (Additional file 1: Table S3).

Our findings suggest that *Ae. albopictus* is likely to become successfully established in Belgium, particularly in the Flanders region and Brussels, under conditions of early and multiple introductions. The GLM analysis of the percentage of successful introductions (Eq. 1), obtained using the dynamAedes models informed with downscaled ERA5Land air temperatures, supports the claim that interannual climatic variability is the dominant factor influencing overwintering success. The northern part of the country appeared more favourable for establishment than the southern part, likely due to warmer climatic conditions. Moreover, both earlier summer introductions and multiple introduction events revealed a positive effect on the probability of successful introduction. However, these scenarios also introduce an additional layer of stochasticity, as the timing and location of the three supplementary introductions are randomly assigned within defined temporal and spatial bounds.

Our results align with those of other studies using different modelling techniques, all indicating that the suitability for *Ae. albopictus* establishment will increase for Belgium in a warming world, becoming significantly higher compared to pre-industrial conditions by the mid-twenty-first century [3, 14–18]. The continuous influx of propagules, i.e., multiple introductions, from neighbouring countries where the species is already established, such as Germany and France, is likely facilitating its establishment in an area at the margins of its current invasive range, as currently observed in Belgium. This dynamic aligns with the source–sink framework described by Pulliam (1988) [19], which distinguishes between source habitats, where local reproduction exceeds mortality, and sink habitats, where populations rely on immigration from source areas to persist. In this case, ongoing immigration appears to play a key role in supporting populations of *Ae. albopictus* in regions where the current local conditions may not otherwise support or favour its persistence.

While our results align with those of other modelling efforts, it is important to emphasise that our approach was designed primarily as an exploration of invasion scenarios rather than a predictive exercise. Within this framework, we focused on a limited set of introduction scenarios, acknowledging the inherent stochasticity of the process. The model relied on life-history traits pooled from the literature, which may not fully reflect recent local adaptations of European *Ae. albopictus* populations [20], and on downscaled ERA5Land air temperatures, which may introduce local biases. These choices are consistent with the goal of identifying plausible dynamics and key drivers of establishment, thereby providing a basis for future surveillance, field validation, and experimental studies.

These results, combined with the fieldwork and citizen science findings, raise the question of whether the eradication of *Ae. albopictus* in Belgium is still feasible, or efforts should shift toward maintaining populations at low densities through control measures to lower nuisance and the risk for local arbovirus transmission upon introduction of viruses. Once populations overwinter in the same locations over multiple years, eradication becomes far more difficult, if not impossible.

Given the current situation, immediate, scalable, and sustainable interventions are essential. While innovative methods such as sterile insect techniques or Wolbachia-based suppression hold promise, they require substantial resources and infrastructure, making them less viable in the short term for Belgium. Instead, integrated vector management approaches are underway, combining active surveillance and citizen science initiatives such as the MosquitoSurveillance app [21] with targeted larval site inspections and longitudinal ovitrap monitoring in areas where overwintering has been documented. Public awareness campaigns to encourage the elimination of breeding sites [22], alongside targeted larviciding using *Bacillus thuringiensis israelensis* (*Bti*) in non-removable water containers, represent practical, immediately deployable strategies. Additionally, the potential of mass trapping of adult mosquitoes as a complementary, non-insecticidal control method warrants further investigation in this context [23].

Looking ahead, the development of scalable and operational modelling tools, co-designed with vector control and surveillance stakeholders and public health authorities, will be essential in ensuring that model predictions truly address field needs [24]. Models capable of forecasting key seasonal dynamics, such as the onset, peak, and offset of *Ae. albopictus* activity, could significantly enhance the timing and efficiency of control efforts [25]. However, to be effective, these tools must go beyond replicating what field scientists already anticipate through prior knowledge and experience. Instead, they should provide actionable insights that complement on-the-ground expertise, offering early warnings, identifying unexpected risk periods, and supporting adaptive management strategies [25].

Beyond its ecological, societal, and public health implications, the establishment of *Ae. albopictus* in Belgium marks a shift to a new epidemiological reality: one where mosquito-borne viruses not previously transmitted by native species could find a foothold. While the current climate may only support transmission during short temporal windows, such as particularly warm summers, a ‘perfect storm’ scenario—where an infected traveller arrives in an area with active mosquito populations during favourable conditions—remains a concern. Further establishment of this vector, coupled with climate change, will likely extend these transmission windows, increasing the probability of local outbreaks. However, these windows will likely remain narrower in Belgium than in Mediterranean regions. Nevertheless, proactive surveillance and targeted control efforts are critical to limiting future risks and protecting public health.

## Supplementary Information

Supplementary material 1.

## Data Availability

Observational data on *Aedes albopictus* collected in Belgium were obtained from the MEMO and MEMO + projects. Temperature data were obtained from the ERA5Land dataset and downloaded from the Climate Data Store ([https://cds.climate.copernicus.eu/#!/home] (https://cds.climate.copernicus.eu/)). All the analyses were performed in R 4.3.3, the codes used are available on GitHub at [https://github.com/danddr/alboBelgium#] (https://github.com/danddr/alboBelgium).
