# Supplementary material for: A warming welcome? Belgium’s increasing suitability for Aedes albopictus
Source: Parasit Vectors. 2025 Nov 26;18:491. doi: 10.1186/s13071-025-07119-w (PMC12659590; doi:10.1186/s13071-025-07119-w)
Supplement: Supplementary file 1 — Supplementary material 1. [file 13071_2025_7119_MOESM1_ESM.docx]

# **A Warming Welcome? Belgium’s Increasing Suitability for *Aedes albopictus***

Daniele Da Re^1,2^, Isra Deblauwe^3^, Emmanuelle Inès Kern^4^, Marie Hermy^5^, Javiera Rebolledo Romero^5^, Katrien Tersago^6^, Veerle Versteirt^7^, Birgit Dumez^8^, Cyrelle Houtsaeger^9^, Lieze Rouffaer^9^, Olivier Beck^10^, Wim Van Bortel^3,11*^

## Supplementary Information

**Table S1** Overview of the introduction scenarios

| **Introduction** | **Date of the introduction** | **Number of eggs** | **Years** |
| --- | --- | --- | --- |
| Single | June 30 | 500, 1000 | 2018-2021 |
| Single | July 15 | 500, 1000 | 2018-2021 |
| Multiple | First introduction event on June 30, then three random events of introduction until 15th September | 500, 1000 | 2018-2021 |
| Multiple | First introduction event on July 15, then three random events of introduction until 15th September | 500, 1000 | 2018-2021 |

**Table S2** Results of ANOVA assessing the effects of season, year, region, and their interactions on air temperature across the three regions of Belgium (Brussels, Flanders, and Wallonia) and years (2018-2021). All factors and interactions showed statistically significant contributions (p < 0.001), indicating pronounced spatiotemporal variability in air temperature.

| **Source** | **Df** | **Sum Sq** | **Mean Sq** | **F value** | **Pr(>F)** |
| --- | --- | --- | --- | --- | --- |
| season | 3 | 439343549 | 146447850 | 7659275 | <2e-16 |
| year | 4 | 5763510 | 1440877 | 75358 | <2e-16 |
| Region | 2 | 5566167 | 2783084 | 145556 | <2e-16 |
| season×year | 12 | 4939396 | 411616 | 21528 | <2e-16 |

**Table S3** Post-hoc Tukey pairwise comparisons of air temperature across seasons, years, and regions. The analysis highlights significant temperature differences within each season and between regions and years, further confirming the spatial and temporal heterogeneity observed in the ANOVA results. DJF: December-January-February; MAM: March-April-May; JJA: June-July-August; SON: September-October-November.

| **Comparison** | **Estimate** | **Lower CI** | **Upper CI** | ***P*-value** |
| --- | --- | --- | --- | --- |
| DJF:2019-DJF:2018 | 0.5351 | 0.5089 | 0.5613 | <0.0001 |
| DJF:2020-DJF:2018 | 2.1619 | 2.1357 | 2.188 | <0.0001 |
| DJF:2021-DJF:2018 | 0.0099 | -0.0163 | 0.0361 | <0.0001 |
| DJF:2022-DJF:2018 | 0.6373 | 0.6111 | 0.6635 | <0.0001 |
| JJA:2019-JJA:2018 | -0.3912 | -0.4171 | -0.3653 | <0.0001 |
| JJA:2020-JJA:2018 | -0.9506 | -0.9765 | -0.9247 | <0.0001 |
| JJA:2021-JJA:2018 | -2.0691 | -2.095 | -2.0432 | <0.0001 |
| JJA:2022-JJA:2018 | 0.0898 | 0.0639 | 0.1157 | <0.0001 |
| MAM:2019-MAM:2018 | -1.1424 | -1.1683 | -1.1165 | <0.0001 |
| MAM:2020-MAM:2018 | -0.54 | -0.5659 | -0.514 | <0.0001 |
| MAM:2021-MAM:2018 | -3.1447 | -3.1706 | -3.1188 | <0.0001 |
| MAM:2022-MAM:2018 | -0.3323 | -0.3582 | -0.3064 | <0.0001 |
| SON:2019-SON:2018 | -0.2984 | -0.3244 | -0.2723 | <0.0001 |
| SON:2020-SON:2018 | 0.6357 | 0.6097 | 0.6618 | <0.0001 |
| SON:2021-SON:2018 | 0.0713 | 0.0453 | 0.0974 | <0.0001 |
| SON:2022-SON:2018 | 1.6956 | 1.6695 | 1.7216 | <0.0001 |
| DJF:2020-DJF:2019 | 1.6268 | 1.6006 | 1.6529 | <0.0001 |
| DJF:2021-DJF:2019 | -0.5252 | -0.5514 | -0.499 | <0.0001 |
| DJF:2022-DJF:2019 | 0.1022 | 0.076 | 0.1284 | <0.0001 |
| JJA:2020-JJA:2019 | -0.5594 | -0.5853 | -0.5335 | <0.0001 |
| JJA:2021-JJA:2019 | -1.678 | -1.7039 | -1.6521 | <0.0001 |
| JJA:2022-JJA:2019 | 0.481 | 0.4551 | 0.5069 | <0.0001 |
| MAM:2020-MAM:2019 | 0.6025 | 0.5766 | 0.6284 | <0.0001 |
| MAM:2021-MAM:2019 | -2.0023 | -2.0282 | -1.9764 | <0.0001 |
| MAM:2022-MAM:2019 | 0.8101 | 0.7842 | 0.836 | <0.0001 |
| SON:2020-SON:2019 | 0.9341 | 0.9081 | 0.9602 | <0.0001 |
| SON:2021-SON:2019 | 0.3697 | 0.3437 | 0.3958 | <0.0001 |
| SON:2022-SON:2019 | 1.994 | 1.9679 | 2.02 | <0.0001 |
| DJF:2021-DJF:2020 | -2.152 | -2.1781 | -2.1259 | <0.0001 |
| DJF:2022-DJF:2020 | -1.5246 | -1.5507 | -1.4984 | <0.0001 |
| JJA:2021-JJA:2020 | -1.1186 | -1.1445 | -1.0926 | <0.0001 |
| JJA:2022-JJA:2020 | 1.0404 | 1.0145 | 1.0663 | <0.0001 |
| MAM:2021-MAM:2020 | -2.6048 | -2.6307 | -2.5789 | <0.0001 |
| MAM:2022-MAM:2020 | 0.2076 | 0.1817 | 0.2335 | <0.0001 |
| SON:2021-SON:2020 | -0.5644 | -0.5905 | -0.5384 | <0.0001 |
| SON:2022-SON:2020 | 1.0598 | 1.0338 | 1.0859 | <0.0001 |
| DJF:2022-DJF:2021 | 0.6274 | 0.6012 | 0.6536 | <0.0001 |
| JJA:2022-JJA:2021 | 2.1589 | 2.133 | 2.1848 | <0.0001 |
| MAM:2022-MAM:2021 | 2.8124 | 2.7865 | 2.8383 | <0.0001 |
| SON:2022-SON:2021 | 1.6243 | 1.5982 | 1.6503 | <0.0001 |
